# Transient Expression of Lumbrokinase (PI239) in Tobacco* (Nicotiana tabacum)* Using a Geminivirus-Based Single Replicon System Dissolves Fibrin and Blood Clots

**DOI:** 10.1155/2017/6093017

**Published:** 2017-08-28

**Authors:** Alexia Dickey, Nan Wang, Edwin Cooper, Lauren Tull, Drew Breedlove, Hugh Mason, Dehu Liu, Kevin Yueju Wang

**Affiliations:** ^1^Department of Natural Sciences, Northeastern State University, Broken Arrow, OK, USA; ^2^Biotechnology Research Institute, Chinese Academy of Agricultural Sciences, Beijing, China; ^3^Laboratory of Comparative Neuroimmunology, Department of Neurobiology, David Geffen School of Medicine, University of California, Los Angeles, Los Angeles, CA, USA; ^4^Biodesign Institute and School of Life Sciences, Arizona State University, Tempe, AZ, USA

## Abstract

Lumbrokinases, a group of fibrinolytic enzymes extracted from earthworm, have been widely used to prevent and treat various cardiovascular diseases. They specifically target fibrin to effectively degrade thrombi without major side effects. Plant expression systems are becoming potential alternative expression platforms for producing pharmaceutical proteins. In this work, a lumbrokinase (PI239) was produced from a plant system. Both wild-type (WT) and plant codon-optimized (OP)* PI239* gene sequences were synthesized and cloned into a geminivirus-based single-vector DNA replicon system. Both vectors were independently expressed in tobacco* (Nicotiana tabacum)* leaves transiently by agroinfiltration. Overexpressed PI239 resulted in sudden tissue necrosis 3 days after infiltration. Remaining proteins were purified through His-tag affinity chromatography and analyzed with SDS-PAGE and Western blot methods. Purified PI239 successfully degraded artificial fibrin with relative activity of 13,400 U/mg when compared with commercial lumbrokinase product.* In vitro *tests demonstrated that plant-derived PI239 dissolved human blood clots and that the plant expression system is capable of producing functional PI239.

## 1. Introduction

Cardiovascular diseases (CVDs) are now the leading cause of death worldwide, causing 1 out of every 3 deaths, resulting in nearly 17.7 million fatalities in 2015. Together, coronary heart disease (7.4 million) and strokes (6.7 million) represented 79.66% of all deaths from CVDs [1]. In today's world, the modern fast-paced lifestyle, unhealthy habits such as an unhealthy diet, tobacco use, alcohol abuse, and lack of exercise, leads to increased deaths. Over 75% of deaths by CVDs were from low- and middle-income countries due to shortages of medical providers and less access to health care services [1]. CVDs therefore represent heavy financial burden on the global economy.

Anticoagulants, such as Heparin and Warfarin, are often used to treat people with established CVDs. Although they are capable of effectively lowering blood viscosity and preventing the formation of new clots, they are not able to degrade existing blood clots. Overdoses of those anticoagulants often result in excessive bleeding and may sometimes be fatal [2]. Lumbrokinases (LKs), a group of fibrinolytic enzymes extracted and purified from earthworm, have attracted researchers' interests [3–5]. These enzymes can reduce platelet aggregation and effectively degrade thrombi. They also decrease blood viscosity and inhibit clot formation. Unlike other thrombolytic agents, such as tissue plasminogen activator (t-PA), streptokinase, and urokinase (u-PA), LKs are specifically targeted to fibrin. As a result, this reduces the risk of serious excessive bleeding and consequently minimizes the probability of fatalities. LKs have been widely used as food supplements to prevent and treat CVDs in Asia, Europe, and North America. Lumbrokinase has been fully studied as an oral fibrinolytic enzyme [6]. In addition, it has also been used in clinical practices in China to treat coronary artery disease, heart attack, stroke, and so forth [3–5]. DLBS1033, a bioactive protein fraction containing mainly LKs extracted from* Lumbricus rubellus* [6], is currently undergoing clinical trials for effects on human fibrinolytic and coagulation system on healthy subjects (Phase 2, completed; ClinicalTrials.gov identifier: NCT01694537) and for the treatment of acute ischemic stroke patient (Phases 2 and 3; ClinicalTrials.gov identifier: NCT02133521). Completion of Phase 4 trial (ClinicalTrials.gov identifier: NCT01865474) for reducing fibrinogen level of diabetes mellitus patients represents a major step forward for the whole field of complementary and alternative medicine. However, conventional extraction and purification methods of commercial LK products from crude pulverized earthworm extract are tedious and complicated. Different extraction and purification methods often result in various enzyme fractions. The final LK products always contain earthworm contaminants (Figure 3(a)), which often induce side effects, such as skin itching, diarrhea, and nausea [5].

Various LK genes have been cloned and produced in different expression systems including* Escherichia coli (E. coli)* [7, 8], yeast [9, 10], and mammalian [11] systems. However, results from those investigations have not been promising. Specifically, recombinant proteins showed either low yield, lack of posttranslational modification (PTM), or low enzymatic activity. Conversely, plant expression systems show great advantages over other platforms. Plants are capable of producing large quantities of recombinant proteins that are free of human pathogens. Additionally, plant systems also enable PTMs which can impact protein behavior and increase protein function [12, 13]. To our knowledge, there are only three previous reports that have used plant systems to produce LKs [14–16]. Ko et al. [16] introduced a LK gene from the earthworm* Eisenia andrei* into tobacco plants. Although high levels of mRNA expression were observed, no enzymatic activity was detected in that study. In 2014, research group successfully expressed a biologically active antithrombotic LK, EFE 3-1 from* L. rubellus*, in sunflower seed kernels [14, 15]. The recombinant LKs yield at 5.1 [14] and 5.42 g/kg [15] in recombinant sunflower seeds, respectively.

For unknown reasons, the results of recombinant LKs derived from different expression systems are not always consistent [5, 11]. We successfully cloned, expressed, and characterized a LK gene,* CST1* from* Eisenia fetida*, in an* E. coli* system and confirmed high antithrombolic activity for recombinant CST1 [7]. However, when we introduced CST1 into transgenic tobacco plants, we did not observe enzymatic activity from the purified CST1 (data now shown). In this study, we chose lumbrokinase PI239 as a candidate gene which was cloned from* Lumbricus bimastus*. This LK has been previously shown to have excellent antithrombotic characteristics in both* E. coli* [8] and yeast [10] systems. Both wild-type (WT) and codon-optimized (OP)* PI239* were cloned into a geminivirus-derived single DNA replicon system.* PI239* was driven by a dual enhanced tobacco mosaic 35S promoter and transiently expressed in the tobacco leaf system. Purified plant-based recombinant PI239 (rPI239) showed fibrin degradation and blood-clot lysis activity. This study demonstrated that the plant system is capable of producing functional rPI239. Overexpression of rPI239 was observed to be toxic to leaf tissue. Potential alternative platforms for producing a cost-effective single lumbrokinase component were discussed.

## 2. Materials and Methods

### 2.1. Plant Expression Constructs

A plant-optimized PI239 DNA (op-PI239) sequence, based upon the GenBank (accession number: AF433650), was designed by OptimumGene™ (GenScript Inc., New Jersey, USA). The optimized sequence was submitted to GenBank (accession number: KT225467). A plant codon-optimized murine monoclonal antibody heavy chain signal peptide (LPH) was fused to N-terminal of mature codon-optimized PI239 (OP) for targeting of translating ribosomes to the ER and subsequent secretion of recombinant proteins into the extracellular space. A hexahistidine tag (His6) and tetrapeptide signal (KDEL) was linked to the C-terminus of both WT-PI239 (full-length PI239 with its native signal peptide) and OP-PI239 (LPH with mature codon-optimized PI239), respectively, to create WT- and OP-PI239-His6-KDEL fragments. KDEL, endoplasmic reticulum (ER) retention signal, was used to enhance foreign protein accumulation in ER. The restriction site, KpnI, was added to the 5′ end of OP-PI239-His6-KDEL, and an XbaI site was linked to the 5′ end of WT-PI239-His6-KDEL. Two additional sites, SacI and BamHI, were created at the 3′ end of both fragments, respectively.

To produce plant expression constructs, both wild-type (WT) and plant-optimized fragment (LPH-OP) sequences were synthesized and cloned into the pUC57 vector by GenScript Inc. to derive the cloning vectors, pUC-WT-Lum and pUC-LPH-Lum. The fragment, WT-PI239-His6-KDEL, was isolated from pUC-WT-Lum via KpnI/SacI and cloned into a geminivirus-derived single replicon construct, pBYR2fp, to produce the transient expression vector, pBYR-WT-Lum. XbaI and SacI restriction enzymes were used to cut the OP-PI239-His6-KDEL fragment from pUC-LPH-Lum and insert it into pBYR2fp to derive pBYR-LPH-OP-Lum for subsequent transient expression assays in plants.

### 2.2. Plant Leaf Agroinfiltration

Two plant expression constructs, pBYR-WT-Lum and pBYR-LPH-OP-Lum, were transformed into* Agrobacterium tumefaciens* LBA4404 by electroporation with a Multiporator (Eppendorf, Hamburg, Germany), respectively. The resulting strains were spread evenly onto the selective LB plates containing kanamycin antibiotic (50 mg/L). After 2-day incubation at 28°C in the dark, the bacteria layer was collected and resuspended in an infiltration buffer as described by Huang et al. [17, 18].* A. tumefaciens* (OD_600_ = 0.5) suspensions were used to infiltrate the abaxial surface of 6 to 10-week-old tobacco plant leaves (*N. tabacum* cv Petite Havana SR1) with a 20 mL syringe (no needle). Infected leaf tissue samples were harvested on day 3, 72 h after infiltration (concurrent with necrosis), for protein extraction and purification.

### 2.3. Electrolyte Leakage Assay

Tissue necrosis was quantified by the electrolyte leakage (EL) method as previously described [19]. A 5 mm cork borer was used to punch leaf discs from an area that was infiltrated with* Agrobacterium tumefaciens*. Leaf discs were briefly rinsed with deionized water (DI) to remove* A. tumefaciens *solution residue. Subsequently, the disc was then floated on 30 mL of DI water in a 25 × 200 mm test tube with continuous shaking at 200 rpm at room temperature. After 1 hr, the EL value (*T*0) was measured with a conductivity meter (VWR Radnor, PA, USA). Sample tubes were then incubated at 98°C in a water bath for 2 hrs to release all electrolytes (*T*1). The level of tissue necrosis was determined by the difference between EL values at* T*0 and* T*1 as a percentage of total conductivity (% EL): 100 × (*T*1 − *T*0)/*T*1. Means ± SE (*n* = 6) were analyzed with a one-way ANOVA test.

### 2.4. SDS-PAGE, Western Blot, and Deglycosylation

Total soluble leaf protein was extracted with the P-PER Plant Protein Extraction Kit with 0.1% Halt™ Protease Inhibitor Cocktail (Thermo Fisher Scientific, Carlsbad, CA, USA) according to the manufacturer's protocol. Approximately 200 *μ*L of protein extract was mixed with 1 mL of Ni-NTA agarose (Qiagen, Venlo, Netherlands) that was equilibrated with Buffer A (50 mM NaH_2_PO_4_, 300 mM NaCl, pH 8.0) and incubated at 4°C on a rocker for 1 hr. The mixture was then added to a 1 mL polypropylene column that was preequilibrated with 1 mL of Buffer B (50 mM NaH_2_PO_4_, 300 mM NaCl, 5 mM imidazole, pH 8.0). Afterwards, the mixture was washed with 10 mL of Buffer A and subsequently with 5 mL of Buffer B by gravity flow. The purified His-tagged protein from agroinfiltrated leaves was eluted with elution buffer (50 mM NaH_2_PO_4_, 300 mM NaCl, 1 M imidazole, pH 8.0). The same steps were performed for nonagroinfiltrated leaf (−CK). The final elution samples from both treated and nontreated leaves were used for subsequent assays. A Bradford assay was performed using a Bradford kit (Bio-Rad, Hercules, CA, USA) for quantification of the purified recombinant proteins.

Purified His-tagged protein was denatured at 95°C in Laemmli Sample Buffer (Bio-Rad) with 2.5% of *β*-mercaptoethanol for 5 min and separated on a precast 12% gel for 1 hr. The protein band was either stained with Bio-Safe™ Coomassie G-250 Stain (Bio-Rad) for visualization or transferred to iBlot® nitrocellulose membrane with an iBlot Gel transfer Device (Thermo Fisher). Relative band density on SDS-PAGE was analyzed with Image Studio (LI-COR Biosciences, Lincoln, Nebraska, USA). The yield of recombinant PI239 was determined based on the Bradford assay and the percentage of total density on SDS gels. Leaf-derived protein eluates were concentrated with Amicon Ultra 10 K spin columns (Sigma-Aldrich, St. Louis, Missouri, USA) and prepared to a concentration of 1 *μ*g/*μ*L for subsequent fibrin plate and blood-clot lysis assays. His-tagged proteins were detected with the HisProbe-HRP Reagent and Kit (Thermo Fischer Scientific) according to the manufacturer's instructions. The membrane was incubated in SuperSignal® Working Solution and exposed to CL-XPosure Film (Thermo Fischer Scientific).

For N-glycan analysis, glycans were released from 10 *μ*g purified recombinant PI239 by PNGase F (Promega, Madison, WI, USA) digestion using denaturing conditions for SDS-PAGE following manufacturer's instructions.

### 2.5. Fibrinolytic Activity Assay

Fibrinolytic activity of LK was analyzed by a modified fibrin plate assay as previously described [7]. Agarose (0.2 g) was dissolved in 40 mL of 1x Phosphate Buffered Saline (PBS) by boiling in a microwave and then cooled to approximately 40°C. Prior to the solidification of the gel, 32 mg of human fibrinogen (Sigma-Aldrich), 10 units of human plasminogen (rPeptide LLC, Bogart, GA, USA), and 10 units of human thrombin (BioPharm Laboratories LLC, Riverton, UT, USA) were added to the gel and mixed with gentle swirling to prevent the introduction of bubbles. The mixture was then poured to Petri dishes and allowed to solidify at room temperature. Wells (3 mm in diameter) were subsequently made in the solidified gel. 10 *µ*L of concentrated samples (1 *µ*g/*µ*L) was diluted in 40 *µ*L of 1x PBS buffer, loaded in the wells of the gels, and incubated at room temperature overnight. Standard lumbrokinase (Doctor's Best Inc., Irvine, CA, USA) was used as positive control (10 *µ*g) and elution samples from nonagroinfiltrated leaves were used as negative control. Actual diameters (mm) of clear halo circles were measured. The fibrinolytic activity of rPI239 was calculated in comparison to the standard LK product (18,000 U/mg) as described in our previous study [7]. Means ± SE (*n* = 4) were analyzed by a one-way ANOVA test.

### 2.6. Blood-Clot Lysis Assay

An* in vitro* blood-clot lysis assay was performed as previously described [7, 20] with minor modifications. Whole human blood (Interstate Blood Bank, Chicago, IL, USA) was sliced to small pieces and washed thoroughly in 1x PBS buffer. Subsequently, blood clots (around 50 mg/clot) were then transferred into a 24-well plate and rinsed two times with 1x PBS buffer. 40 *µ*L of concentrated samples (1 *µ*g/*µ*L) was diluted in 460 *µ*L of 1x PBS buffer and added to the wells containing treated blood clots. After incubation at 37°C overnight, undissolved samples were measured and weight differences were determined for samples before and after treatment. PBS buffer and elution samples from nontransfected leaves were used as negative controls. Standard LK (40 *µ*g) was used as a positive control. The clot lysis was calculated as a percentage of weight difference/untreated sample. Means ± SE (*n* = 3) were analyzed by a one-way ANOVA test.

## 3. Results and Discussion

Plant expression systems provide advantages for pharmaceutical production in terms of safety, speed, and cost-effectiveness [12, 13]. LKs produced from* E. coli*, yeast, or animal systems show either the formation of inclusion bodies, low yield, or partial activity. Due to these complications, LK genes are good candidates for expression in plant platforms. Meanwhile, LK products have been used as health supplements worldwide. Future fruit- or vegetable-derived LKs could be used directly for oral administration to fight against CVDs without further downstream purification processes [5]. To date, over 28 LK gene sequences have been deposited in Genbank; only EFE3-1 was expressed and characterized in plant (sunflower seeds) [14, 15]. Here, we show the successful production of a functional LK (PI239) in tobacco leaves via a geminivirus-derived single DNA replicon system.

### 3.1. Geminivirus-Based Plant Transient Expression Vectors

**Figure 1:**
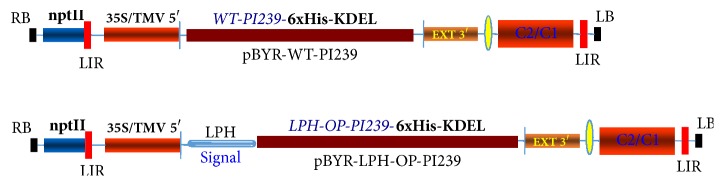
*Diagrams of the vectors (pBYR-WT-PI239 and pBYR-LPH-OP-PI239) used in this study*. 35S/TEV 5′, CaMV 35S promoter with tobacco mosaic virus 5′ UTR; EXT 3′, tobacco gene extension gene 3′ element; nptII, expression cassette encoding nptII gene for kanamycin resistance; LIR, long intergenic region of BeYDV genome; SIR, short intergenic region of BeYDV genome; C1/C2, BeYDV ORFs C1 and C2, encoding Rep and RepA; LB and RB, the left and right borders of the T-DNA region; 6xHis, hexahistidine tag for Ni-NTA affinity purification; KDEL, ER retention peptide; WT, full-length PI239 with its native signal; LPH-OP, a plant codon-optimized murine monoclonal antibody heavy chain signal peptide (LPH) that was fused to the N-terminal of mature codon-optimized PI239 (OP) sequence.

Various studies have demonstrated that this replicon system can efficiently produce high-level expression of foreign therapeutic proteins. It has been used to produce ZMapp antibodies, the only effective drug for Ebola virus, from tobacco leaves [18, 21]. It is known that codon optimization of a gene sequence can increase foreign protein expression [22]. In this work, we optimized the PI239 gene codon for tobacco preferences without changing its primary amino acid sequence. After codon optimization, the codon adaptation index (CAI) was upgraded from 0.69 to 0.86 and the GC content was reduced from 54.02% to 42.65% (GenBank accession number: KT22546). A plant-optimized LPH signal was fused to the N-terminal of mature PI239 (OP). LPH is a secretory signal and can target recombinant proteins into the apoplast and increase protein yield [23]. Reliable His_6_-tag affinity purification will improve rPI239 purification process. KDEL is endoplasmic reticulum (ER) retention signal that enables increased accumulation of foreign proteins, prevents the degradation of recombinant proteins, and modifies the glycosylation process [13, 23]. Therefore, a His_6_-KDEL peptide was appended to the C-terminus of both WT-PI239 and OP-PI239 in order to enhance protein expression. Two geminivirus-based plant expression vectors, pBYR-LPH-OP-Lum and pBYR-WT-Lum, were cloned as described in Materials and Methods. After completion of the constructs, digestion with specific restriction enzymes confirmed that the gene fragments were intact (Figure 1).

### 3.2. Overexpression of PI239 Causes Tissue Necrosis

**Figure 2:**
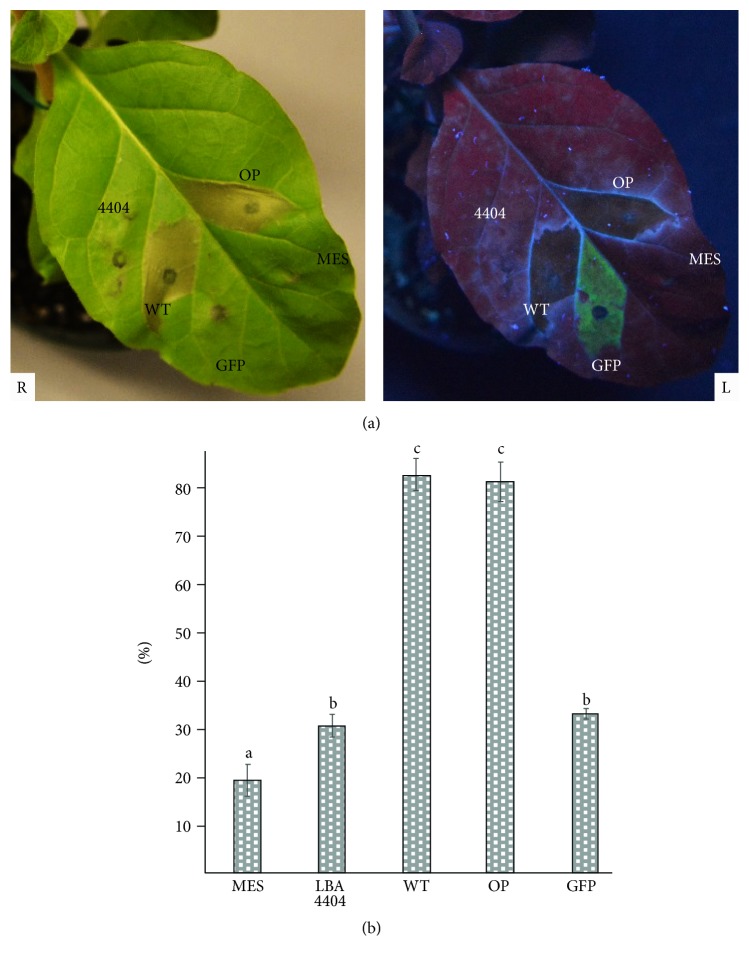
*Expression of PI239 in leaves resulted in necrosis 3 days after infiltration*. (a) Leaf necrosis (R) and under the UV light (L) of a hand-held lamp. (b) Electrolyte leakage assay for membrane damage. GFP: pBY030.2R (geminivirus-based single replicon system containing green fluorescence protein); 4404:* A. tumefaciens *LBA4404 strain only; MES: infiltration solution only; WT: pBYR-WT-PI239; OP: pBYR-LPH-OP-PI239. Values represent the means ± SE. Different letters indicate significant differences (*p* ≤ 0.05; *n* = 6; one-way ANOVA test).

Proteases can hydrolyze peptide bonds and breakdown proteins. It is very important to balance cell divisions, maintain normal growth, and regulate development in life [24–26]. Protease-induced cell death (apoptosis) has been extensively investigated in animal systems [24]. It is plausible that plants also share similar protease-induced programmed cell death [26]. The information pertaining to how and why protease activity leads to cell death in plants is still very limited [24, 26]. One report indicated that enhanced native protease, Cf-4, causes plant leaf tissue necrosis [27]. In this study, expressed foreign recombinant protease from earthworm also resulted in cell death. Our results showed that the overexpression of PI239 in plant leaves induced apoptotic cell death (Figure 2).

Three days after infiltration, leaf areas of* Agrobacteria*-infiltrated pBYR-LPH-OP-Lum and pBYR-WT-Lum showed sudden chlorosis and produced extensive tissue necrosis (Figure 2(a)). When plants were infiltrated with a positive control plasmid for GFP expression (pBY030.GFP), dim green fluorescence was observed in infiltrated tissue. It is important to note that no apparent tissue damage was observed from leaves that were infiltrated with* tumefaciens *LBA4404, infiltration solution (MES), or pBY030.GFP alone. Conductivity measurements indicated that foreign PI239 accumulation induced membrane damage and ion leakage (Figure 2(b)). Infiltration solution (MES),* A. tumefaciens *LBA4404, or pBY030.GFP showed EL damage at 19%, 23%, or 32%; respectively, most likely because* Agrobacterium* infiltration or the infiltration process itself causes slight damage to the leaf tissue. Conversely, the electrolyte leakage that was observed in leaves transfected with pBYR-LPH-OP-Lum and pBYR-WT-Lum was 83% and 81%, respectively. No significant differences (*p* ≤ 0.05) in tissue death or membrane leakage differences were observed between leaves that were transiently expressing pBYR-LPH-OP-Lum and pBYR-WT-Lum.

Perhaps PI239 might hydrolyze native proteins in plants, including membrane proteins, resulting in irreversible membrane disruption, permeability, and cell death. This may also explain why some LK genes were capable of being produced in* E. coli* but not in other systems. Since recombinant LKs are expressed and aggregated in inclusion bodies, they lose capability for folding properly with correct structural configurations and, therefore, are unable to hydrolyze membranes and kill* E. coli* host cells. Previous attempts to express functional LKs in animal cells [11] or plant cells were not uniformally successful [16]. The aforementioned systems are able to process posttranslational modifications and produce functional LKs which may inhibit the growth of transformed cells, perhaps explaining why the majority of expressed LKs are either nonfunctional or less active. A plant seed-derived LK (EFE3-1) showed strong antithrombotic activity [14, 15]. Seeds have low water content, a reduced acidic environment, and greater abundance of protease inhibitors. Collectively, these factors inhibit protease activity and thereby allow seeds to produce intact and functional LK [14, 15]. In our unpublished studies, additional blood-clot-dissolving proteases expressed transiently in leaves also resulted in tissue death (data in preparation). Understanding precisely how these clot-dissolving proteases affect plant cell death is worthy of further investigation.

### 3.3. Recombinant PI239 Purification and Analysis

**Figure 3:**
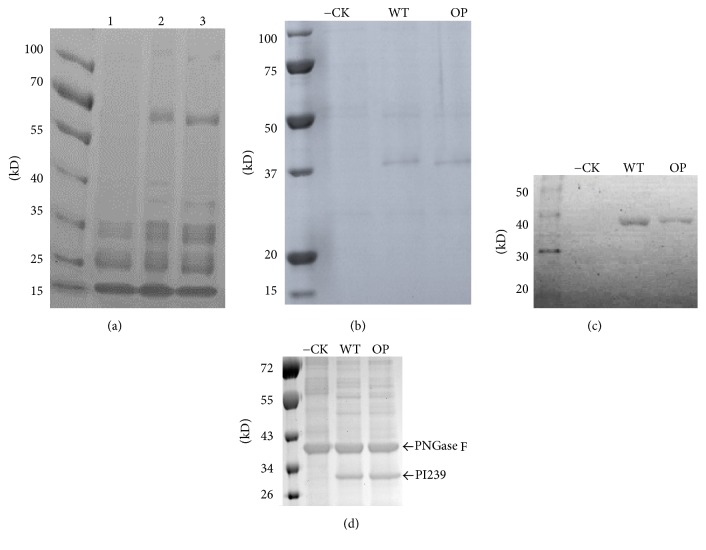
*SDS-PAGE of commercial LK product (a), SDS-PAGE (b), Western blot (c), and deglycosylation (d) analysis of rPI239*. Lanes 1, 2, and 3 are LK product from Doctor's Best, Boluke, and Nutricology. −CK: negative control from a noninfiltrated leaf. WT: pBYR-WT-PI239; OP: pBYR-LPH-OP-PI239; M: protein molecular weight marker.

Commercial LK product is very impure and contains lots of native proteins from earthworm (Figure 3(a)). His-tagged rPI239 was purified with a Ni-NTA agarose affinity chromatography matrix and separated with SDS-PAGE (Figure 3(b)). As shown on the gel, a band with an estimated molecular weight of ~37 kDa was observed in both pBYR-LPH-OP-Lum and pBYR-WT-Lum lanes. No corresponding band was evident in the noninfiltrated control lane. In addition, the ~37 kDa band was also detected by Western blot assay via HisProbe-HRP method (Figure 3(c)). The observed molecular weight (~37 kDa) is larger than the predicted size of PI239-His6-KDEL chain (32 kDa) and this discrepancy is most likely due to glycosylation of the PI239 protein. After deglycosylation treatment with PNGase F, the ~32 kDa band was observed (Figure 3(d)). PNGase F catalyzes the cleavage of N-Linked oligosaccharides. Within the PI239 protein, the Asn149 amino acid residue is a known N-glycosylated site [9, 10] and N-linked glycosylation is well known to result in shifting of protein electrophoretic mobility [28]. We fused the ER signal peptide, KDEL, to the C-terminus of rPI239. This sequence is known to increase recombinant protein retention in the ER and enhance accumulation [13]. Glycosylation is initiated in the ER and due to the KDEL sequence. It is probable that the recombinant proteins were indeed glycosylated. ER-based glycosylation can prevent plant-specific xylose and fucose, both of which are known to induce immunogenic responses [13, 29]. Because LKs are produced in the earthworm digestive tract, glycosylation helps LKs' stability and prevents the degradation of LKs by an acidic and proteolytic environment.

A geminivirus-based single replicon system has been used widely to produce high levels of recombinant protein [17, 18, 21]. For example, the yield of mAb against Ebola virus reached 0.5 mg/leaf fresh weight (LFW) using this system with a leaf infiltration procedure for transfection [17]. In this study, the yield of PI239 was low because the overexpression of the PI239 protein resulted in sudden tissue death 3 days after infiltration. The remaining proteins were collected from necrotic tissue and purified with Ni-NTA affinity columns. Averages of 20.1 *µ*g/g leaf fresh weight (LFW) with pBYR-LPH-OP-Lum and 21.3 *µ*g/g LFW with pBYR-WT-Lum were calculated based upon a Bradford assay and the intensity of the bands on SDS-PAGE gel. No significant expression differences between pBYR-WT-Lum and pBYR-LPH-OP-Lum were observed from the SDS-PAGE analysis.

### 3.4. Fibrinolytic and Blood-Clot Lysis Activity of Recombinant PI239

**Figure 4:**
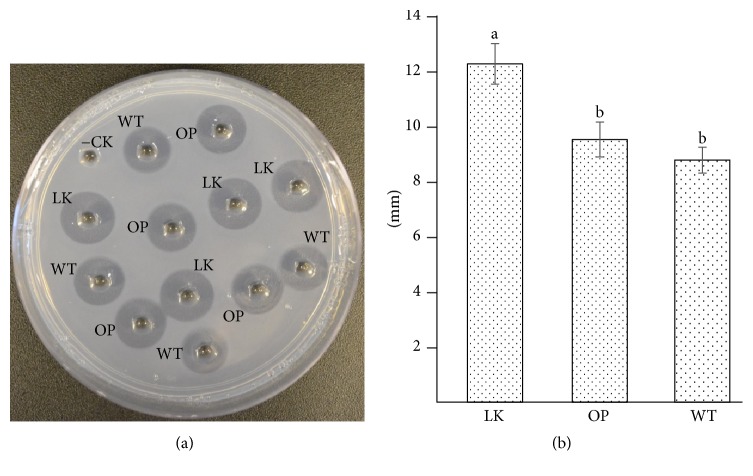
*Fibrin plate assay*. (a) Purified elution was tested on a fibrin plate. (b)* Diameters of lysis halo area*. Well −CK: elution from noninfiltrated leaf (negative control); WT: pBYR-WT-PI239; OP: pBYR-LPH-OP-PI239. LK: standard lumbrokinase (18,000 u/mg). Values represent the means ± SE. Different letters indicate significant differences (*p* ≤ 0.05; *n* = 4; one-way ANOVA test).

LKs are capable of degrading blood clots by targeting fibrin directly. They also can enhance endogenous t-PA activity to convert plasminogen into plasmin, resulting in fibrinolysis. In comparison to other blood-clot-dissolving serine proteases (such as t-PA or u-PA), PI239 shares common features, such as substrate specificity determinants, catalytic subsites, and a catalytic triad; those features are critical for clot breakdown [10]. In this study, a fibrin plate assay demonstrated that plant-derived rPI239 clearly dissolved fibrin (Figure 4). A half-transparent halo area was displayed around both the commercial LK and recombinant protein (Figure 4(a)). The lytic diameters of rPI239 derived from pBYR-LPH-OP-Lum and pBYR-WT-Lum were 9.5 and 8.8 mm. No significant differences were observed between pBYR-LPH-OP-Lum and pBYR-WT-Lum (Figure 4(b)). The activity of rPI239 was lower than commercial LK (18,000 U/mg), whose lytic diameter was 12.25 mm. Relative fibrinolytic activity of rPI239 was calculated to be approximately 13,400 U/mg.

**Figure 5:**
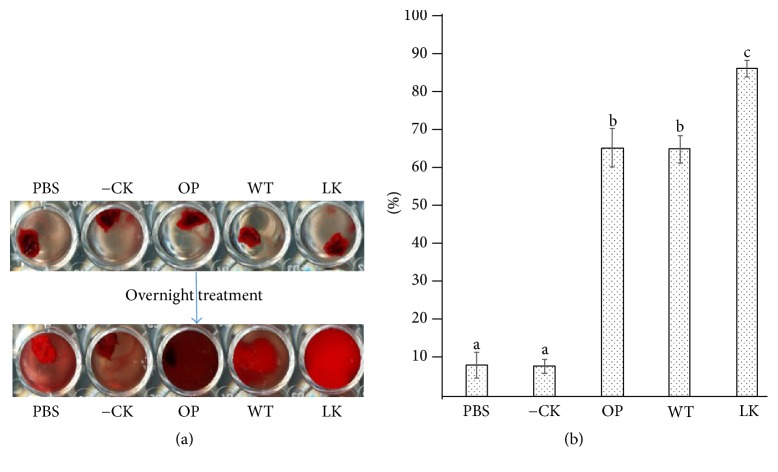
*In vitro human blood-clot lysis assay*. (a) Recombinant PI239 dissolved human blood clot. (b) Percentage of blood-clot lysis. PBS: 1x Phosphate Buffered Saline; −CK: elution from noninfiltrated leaf (negative control); WT: pBYR-WT-PI239; OP: pBYR-LPH-OP-PI239. LK: standard lumbrokinase (18,000 u/mg). Values represent the means ± SE. Different letters indicate significant differences (*p* ≤ 0.01; *n* = 3; one-way ANOVA test).

Plant-derived rPI239 dissolves human blood clots* in vitro* (Figure 5(a)). Slight lysis was observed in the PBS well and noninfiltrated leaf extract well via a clot autolysis process. Significant lysis was observed when clots were treated with standard LK and rPI239. Standard LK, pBYR-LPH-OP-Lum, and pBYR-WT-Lum showed 92%, 73%, and 76% clot lysis, respectively (Figure 5(b)). No significant differences were detected between pBYR-LPH-OP-Lum and pBYR-WT-Lum recombinant proteins. In comparison to standard LK, a lower activity of rPI239 might be due to plant components or residual eluate from the purification process. In a previously reported study, residual plant contaminants present in final elution significantly interfered with the enzymatic activity of another clot-dissolving protease, vampire bat plasminogen activator (DSPA*α*1) [30]. As a result, the purification strategy needs to be further optimized in order to achieve high purity of rPI239, which will effectively result in enhanced fibrinolytic activity.

Guan et al. [14, 15] first produced biologically active LK protein from transgenic sunflower seed kernels. Seed-derived rLK showed high levels of fibrinolytic activity. Mice fed with seed kernels demonstrated antithrombotic effects. In this study, we successfully expressed functional rPI239 transiently in infiltrated tissue. While overexpressed rPI239 killed leaf tissue 3 days after infiltration, purified recombinant protein was confirmed to degrade fibrin and dissolve human blood clots. In this study, we did not see any advantages of codon-optimized PI239 sequences in relative comparison to a wild-type version. The yield of PI239 was also low. It could be explained that PI239 caused necrotic tissue. The proteolytic components released from necrotic tissue degraded most of recombinant PI239. Further study on why LK caused necrosis in plant may find a way to prevent cell death.

## 4. Conclusions: Plant-Derived LK Dissolves Fibrin and Blood Clots

We have demonstrated that a plant system is capable of producing functional rPI239 via a geminivirus-based transient expression system. While this system has been successfully used for the production of various therapeutic proteins, including the well-known ZMapp antibodies for the Ebola virus, it is not suitable for rPI239 production on an industrial scale due to the necrosis that is observed within 3 days as a result of high expression of LK. We have cloned both WT-PI239 and OP-PI239 into a plant binary expression vector and stable plant transformation of PI239 is underway since plants are capable of producing recombinant proteins in large scale. Purified LK can be administered via injection to fight CVDs. LK can also be produced in edible organs, vegetables, or fruits. Because LK can enter the blood stream via the gastrointestinal tract, it is plausible that patients could consume such transgenic vegetables or fruits in an effort to prevent CVDs, thereby eliminating the need for downstream purification processes.
